# Dynamics of Structured Networks of Winfree Oscillators

**DOI:** 10.3389/fnsys.2021.631377

**Published:** 2021-02-10

**Authors:** Carlo R. Laing, Christian Bläsche, Shawn Means

**Affiliations:** School of Natural and Computational Sciences, Massey University, Auckland, New Zealand

**Keywords:** Winfree oscillators, coupled oscillators, neuronal networks, degree, assortativity, copula, Ott/Antonsen

## Abstract

Winfree oscillators are phase oscillator models of neurons, characterized by their phase response curve and pulsatile interaction function. We use the Ott/Antonsen ansatz to study large heterogeneous networks of Winfree oscillators, deriving low-dimensional differential equations which describe the evolution of the expected state of networks of oscillators. We consider the effects of correlations between an oscillator's in-degree and out-degree, and between the in- and out-degrees of an “upstream” and a “downstream” oscillator (degree assortativity). We also consider correlated heterogeneity, where some property of an oscillator is correlated with a structural property such as degree. We finally consider networks with parameter assortativity, coupling oscillators according to their intrinsic frequencies. The results show how different types of network structure influence its overall dynamics.

## 1. Introduction

The behavior of networks of coupled oscillators is a topic of ongoing interest (Strogatz, 2000; Pikovsky et al., 2001; Arenas et al., 2008). While an individual oscillator may have very simple behavior, it is the emergent behavior such as synchronization that has gained much attention (Winfree, 2001; Strogatz, 2003). Networks of coupled oscillators provide insights into physiological systems such as neuronal or cardiac systems, where synchrony or lack thereof can have profound implications (Fenton et al., 2002; Milton and Jung, 2013).

One of the first models for interacting oscillators was the Winfree model (Winfree, 1967; Ariaratnam and Strogatz, 2001; Pazó and Montbrió, 2014; Ha et al., 2015; Gallego et al., 2017; Pazó et al., 2019; Pazó and Gallego, 2020). Each Winfree oscillator is described by a single angular variable and when uncoupled is assumed to undergo periodic oscillations. Each oscillator is assumed to have a phase response curve, a function of its own phase, which can be measured from individual neurons, for example Schultheiss et al. (2011) and Netoff et al. (2005). This describes how an oscillator's phase changes as the result of input from other oscillators. The output from an oscillator is assumed to be in the form of a non-negative pulsatile function of its own phase, and the inputs to an oscillator are assumed to be additive.

A number of authors have studied networks of Winfree oscillators, but as far as we are aware, only in the all-to-all coupled case. Although straightforward to assemble, such networks do not reproduce complex network structures observed in real-world systems such as assortativities between individual neurons (de Santos-Sierra et al., 2014; Teller et al., 2014). We are interested in networks with far more varied structure, not just randomly connected. These networks are *directed*, i.e., edges connect one oscillator to another, without necessarily having a reciprocal connection, as occurs in networks of neurons. There are many ways to create structured networks and here we consider the following: correlating the in- and out-degrees of an oscillator (i.e., the number of inputs and the number of outputs of an oscillator, section 3), inducing degree assortativity (i.e., connecting two oscillators based on their in- and out-degrees, section 4), correlating some local property of an oscillator with either its in- or out-degree (section 5), and inducing parameter assortativity (i.e., connecting two oscillators based on the similarities of an intrinsic property of the two oscillators, such as their free-running frequency, section 6).

Our main tool is the derivation and then numerical analysis of moderately large sets of coupled ordinary differential equations (ODEs). The derivation utilizes the Ott/Antonsen ansatz (Ott and Antonsen, 2008, 2009), an exact technique for dimension reduction in large networks of sinusoidally coupled phase oscillators, of which Winfree oscillators are an example. Some of the computational techniques used here have been presented before (Bläsche et al., 2020; Laing and Bläsche, 2020) but for networks of theta neurons (Ermentrout and Kopell, 1986) rather than Winfree oscillators. In section 2, we present the general model and its reduction using the Ott/Antonsen ansatz. The results are presented in sections 3–6 and we conclude in section 7.

## 2. Model

We consider the network version of the model as presented in Pazó and Montbrió (2014)

(1) $$\begin{array}{l} \frac{d\theta_{j}}{dt}=\omega_{j}+U\left(\theta_{j}\right)\frac{\epsilon }{\langle k\rangle }\overset{N}{\underset{n=1}{\sum }}A_{jn}T\left(\theta_{n}\right) \end{array}$$

for *j* = 1, …*N* where the ω_*j*_ are chosen from a distribution *g*(ω), ϵ is the strength of coupling, 〈*k*〉 is the mean degree of the network, and the connectivity of the network is given by the adjacency matrix *A*, where *A*_*jn*_ = 1 if oscillator *n* connects to oscillator *j* and zero otherwise. The function *U* is known as the phase response curve and we choose it to be

(2) $$\begin{array}{l} U\left(\theta \right)=\sin\beta -\sin\left(\theta +\beta \right) \end{array}$$

so that *U*(0) = 0. If β < π/2 then this function describes a type-II oscillator whereas β = π/2 describes a type-I oscillator (Tsubo et al., 2007). We consider only type-II oscillators in this work. The pulsatile function *T* is given by

(3) $$\begin{array}{l} T\left(\theta \right)=a_{q}{\left(1+\cos\theta \right)}^{q} \end{array}$$

where *q* is a positive integer and $a_{q}=2^{q}{\left(q!\right)}^{2}/\left(2q\right)!$ so that $\int_{0}^{2\pi }T\left(\theta \right)d\theta =2\pi$. The in-degree of oscillator *j* is

(4) $$\begin{array}{l} k_{in,j}=\overset{N}{\underset{n=1}{\sum }}A_{jn} \end{array}$$

and the out-degree of oscillator *n* is

(5) $$\begin{array}{l} k_{out,n}=\overset{N}{\underset{j=1}{\sum }}A_{jn} \end{array}$$

We consider large networks with all oscillators having large in- and out-degrees. Following Chandra et al. (2017) and Laing and Bläsche (2020), we assume that the network can be characterized by two functions: the degree distribution *P*(**k**), where **k** = (*k*_*in*_, *k*_*out*_), and *k*_*in*_ and *k*_*out*_ are the in- and out-degrees of an oscillator, respectively, and an assortativity function *a*(**k**′ → **k**) giving the probability that an oscillator with degree **k**′ connects to one with degree **k**, given that such oscillators exist. Note that we follow (Chandra et al., 2017; Laing and Bläsche, 2020) and normalize *P*(**k**) such that $\underset{\text{k}}{\sum }P\left(\text{k}\right)=N$.

In the limit *N* → ∞ the network is described by the probability density function *f* (θ, ω|**k**, *t*) where *f* (θ, ω|**k**, *t*)*dθ dω* is the probability that an oscillator with degree **k** has phase in [θ, θ + *d*θ] and value of ω in [ω, ω + *d*ω] at time *t*. This function satisfies the continuity equation

(6) $$\begin{array}{l} \frac{\partial f}{\partial t}+\frac{\partial }{\partial \theta }\left(vf\right)=0 \end{array}$$

where

(7) $$\begin{array}{l} v\left(\theta ,\omega ,\text{k},t\right)=\omega +\epsilon U\left(\theta \right)R\left(\text{k},t\right) \end{array}$$

where

(8) $$\begin{array}{l} R\left(\text{k},t\right)=\frac{1}{\langle k\rangle }\underset{{\text{k}}^{\prime }}{\sum }P\left({\text{k}}^{\prime }\right)a\left({\text{k}}^{\prime }\to \text{k}\right)G\left({\text{k}}^{\prime },t\right) \end{array}$$

and

(9) $$\begin{array}{l} G\left({\text{k}}^{\prime },t\right)=\int_{-\infty }^{\infty }\int_{0}^{2\pi }f\left(\theta^{\prime },\omega^{\prime }|{\text{k}}^{\prime },t\right)T\left(\theta^{\prime }\right)d\theta^{\prime }\;d\omega^{\prime } \end{array}$$

The nature of this system [specifically, having *U*(θ) being a single sinusoidal function of θ] means that it is amenable to the use of the Ott/Antonsen ansatz (Ott and Antonsen, 2008, 2009). We assume that

(10) $$\begin{array}{l} g\left(\omega \right)=\frac{\Delta /\pi }{{\left(\omega -\omega_{0}\right)}^{2}+\Delta^{2}} \end{array}$$

where Δ is the half-width-at-half-maximum and ω_0_ the median of the distribution of intrinsic frequencies. Using standard techniques (Chandra et al., 2017; Laing, 2017) which rely on the Ott/Antonsen theory, one can show that the long-time dynamics of the network is described by

(11) $$\begin{array}{l} \frac{\partial b\left(\text{k},t\right)}{\partial t}=\frac{\epsilon e^{-i\beta }R\left(\text{k},t\right)}{2}+\left[i\omega_{0}-\Delta +i\epsilon \sin\beta R\left(\text{k},t\right)\right]b\left(\text{k},t\right)\\ \;-\frac{\epsilon e^{i\beta }R\left(\text{k},t\right)}{2}{\left[b\left(\text{k},t\right)\right]}^{2} \end{array}$$

where

(12) $$\begin{array}{l} G\left(\text{k},t\right)=a_{q}\left[C_{0}+\overset{q}{\underset{j=1}{\sum }}C_{j}\left\{{\left[b\left(\text{k},t\right)\right]}^{j}+{\left[\bar{b}\left(\text{k},t\right)\right]}^{j}\right\}\right] \end{array}$$

where overline indicates complex conjugate and

(13) $$\begin{array}{l} C_{j}=\overset{q}{\underset{k=0}{\sum }}\overset{k}{\underset{m=0}{\sum }}\frac{q!\delta_{k-2m,j}}{2^{k}\left(q-k\right)!m!\left(k-m\right)!} \end{array}$$

The quantity

(14) $$\begin{array}{l} b\left(\text{k},t\right)=\int_{-\infty }^{\infty }\int_{0}^{2\pi }f\left(\theta ,\omega |\text{k},t\right)e^{i\theta }d\theta \;d\omega \end{array}$$

is the complex-valued order parameter for oscillators with degree **k**.

Equation (11) is the general equation describing the dynamics of the network and we use it as a base for analysing a number networks with different types of structure. In section 3, we consider correlations between an individual oscillator's in-degree and its out-degree, as described by the degree distribution *P*(**k**). In section 4, we consider correlations between the degrees of connected oscillators, effectively modifying the function *a*(**k**′ → **k**). In section 5, we investigate the results of one of the parameters intrinsic to an oscillator (ω_0_, Δ, or β) being correlated with a *network* property of that oscillator (its in- or out-degree). Section 6 considers the case when all oscillators have the same in- and out-degrees, and the assortativity function *a*(**k**′ → **k**) is replaced by a function describing the probability of connecting oscillators based on the values of one of their intrinsic parameters—in this case, ω_0_. We conclude in section 7.

## 3. Within Oscillator Correlations

We first consider the effects of correlating an oscillator's in- and out-degree. This general question has been considered by a number of authors studying different types of oscillators (LaMar and Smith, 2010; Vasquez et al., 2013; Martens et al., 2017; Nykamp et al., 2017; Vegué and Roxin, 2019) and experimental evidence for within-neuron degree correlations is given in Vegué et al. (2017). Our derivation follows Laing and Bläsche (2020).

Assuming neutral assortativity we have (Restrepo and Ott, 2014)

(15) $$\begin{array}{l} a\left({\text{k}}^{\prime }\to \text{k}\right)=\frac{k_{out}^{\prime }k_{in}}{N\langle k\rangle } \end{array}$$

where we have assumed that the largest in- and out-degrees are significantly smaller than *N*, so that *a*(**k**′ → **k**) ≤ 1. We will write $P\left(k_{in},k_{out},\hat{\rho }\right)$ instead of *P*(**k**)/*N*, where $\hat{\rho }$ is a parameter controlling the correlation between *k*_*in*_ and *k*_*out*_, explained in detail below. Substituting (15) into (8) we have

(16) $$\begin{array}{l} R\left(k_{in},k_{out},t\right)=\frac{N}{\langle k\rangle }\underset{k_{in}^{\prime }}{\sum }\underset{k_{out}^{\prime }}{\sum }P\left(k_{in}^{\prime },k_{out}^{\prime },\hat{\rho }\right)a\left({\text{k}}^{\prime }\to \text{k}\right)G\left(k_{in}^{\prime },k_{out}^{\prime },t\right)\\ \;=\frac{k_{in}}{{\langle k\rangle }^{2}}\underset{k_{in}^{\prime }}{\sum }\underset{k_{out}^{\prime }}{\sum }P\left(k_{in}^{\prime },k_{out}^{\prime },\hat{\rho }\right)k_{out}^{\prime }G\left(k_{in}^{\prime },k_{out}^{\prime },t\right) \end{array}$$

This is clearly independent of *k*_*out*_, thus *v* must also be independent of *k*_*out*_, the state of an oscillator with degree (*k*_*in*_, *k*_*out*_) must be independent of *k*_*out*_, and thus *G* must be independent of $k_{out}^{\prime }$. So we can write

(17) $$\begin{array}{l} R\left(k_{in},t\right)=\frac{k_{in}}{{\langle k\rangle }^{2}}\underset{k_{in}^{\prime }}{\sum }Q\left(k_{in}^{\prime },\hat{\rho }\right)G\left(k_{in}^{\prime },t\right) \end{array}$$

where

(18) $$\begin{array}{l} Q\left(k_{in}^{\prime },\hat{\rho }\right)\equiv \underset{k_{out}^{\prime }}{\sum }P\left(k_{in}^{\prime },k_{out}^{\prime },\hat{\rho }\right)k_{out}^{\prime } \end{array}$$

Thus, the model equations of interest are

(19) $$\begin{array}{l} \frac{\partial b\left(k_{in}\right)}{\partial t}=\frac{\epsilon e^{-i\beta }R\left(k_{in}\right)}{2}+\left[i\omega_{0}-\Delta +i\epsilon \sin\beta R\left(k_{in}\right)\right]b\left(k_{in}\right)\\ \;-\frac{\epsilon e^{i\beta }R\left(k_{in}\right)}{2}{\left[b\left(k_{in}\right)\right]}^{2} \end{array}$$

where *G* is given by (12) but with the degree dependence being on only *k*_*in*_. Note that the model equations are independent of *N*, the total number of oscillators.

### 3.1. Generating Correlated Degrees

The correlations between an oscillator's in- and out-degree are controlled by the function $P\left(k_{in},k_{out},\hat{\rho }\right)$ and we now describe how to generate these correlations. For simplicity we assume that the distributions of the in- and out-degrees are the same, namely uniform distributions between *m* and *M*, i.e.,

(20) $$p(k)=\{\begin{array}{ll} \frac{1}{M-m} & m\leq k\leq M\\ 0 & \text{otherwise} \end{array}$$

We introduce correlations between the in- and out-degree of an oscillator while retaining these marginal distributions, using a Gaussian copula (Nelsen, 2007). The correlated bivariate normal distribution with zero mean is

(21) $$\begin{array}{l} f\left(x,y,\hat{\rho }\right)=\frac{1}{2\pi \sqrt{1-{\hat{\rho }}^{2}}}e^{-\left(x^{2}-2\hat{\rho }xy+y^{2}\right)/\left[2\left(1-{\hat{\rho }}^{2}\right)\right]} \end{array}$$

where $\hat{\rho }\in \left(-1,1\right)$ is the correlation between *x* and *y*. The variables *x* and *y* have no physical meaning and we use the copula just as a way of deriving an analytic expression for $P\left(k_{in},k_{out},\hat{\rho }\right)$ for which the correlations between *k*_*in*_ and *k*_*out*_ out can be varied systematically. The cumulative distribution function for *x* is

(22) $$\begin{array}{l} C\left(x\right)=\left[1+\text{erf}\left(x/\sqrt{2}\right)\right]/2 \end{array}$$

and the cumulative distribution function for degree *k* is

(23) $$\begin{array}{l} C_{k}\left(k\right)=\int_{m}^{k}\frac{1}{M-m}ds=\frac{k-m}{M-m} \end{array}$$

The joint degree distribution for *k*_*in*_ and *k*_*out*_ is

(24) $$\begin{array}{l} P(k_{in},k_{out},\hat{\rho })={\left\{C^{-1}[C_{k}(k_{in})]\right\}}^{\prime }{\left\{C^{-1}[C_{k}(k_{out})]\right\}}^{\prime }f\{C^{-1}[C_{k}(k_{in})],\\ \;C^{-1}[C_{k}(k_{out})],\hat{\rho }\} \end{array}$$

where the superscript “−1” indicates the inverse of the corresponding function. Now

(25) $$\begin{array}{l} C^{-1}\left[C_{k}\left(k_{in}\right)\right]=\sqrt{2}{\text{erf}}^{-1}\left(\frac{2\left(k-m\right)}{M-m}-1\right) \end{array}$$

and

(26) $$\begin{array}{l} {\left\{C^{-1}\left[C_{k}\left(k_{in}\right)\right]\right\}}^{\prime }=\sqrt{\frac{\pi }{2}}\exp\left[{\left\{{\text{erf}}^{-1}\left(\frac{2\left(k-m\right)}{M-m}-1\right)\right\}}^{2}\right]\frac{2}{M-m}\\ \;=\sqrt{\frac{\pi }{2}}\frac{2}{M-m}\exp\left[\frac{{\left(C^{-1}\left[C_{k}\left(k_{in}\right)\right]\right)}^{2}}{2}\right] \end{array}$$

So

(27) $$\begin{array}{l} P(k_{in},k_{out},\hat{\rho })=\frac{1}{{\left(M-m\right)}^{2}\sqrt{1-{\hat{\rho }}^{2}}}\exp\left\{\frac{\hat{\rho }C^{-1}[C_{k}(k_{in})]C^{-1}[C_{k}(k_{out})}{1-{\hat{\rho }}^{2}}\right\}\\ \;\times \exp\left[\frac{-{\hat{\rho }}^{2}\left({\left\{C^{-1}\left[C_{k}\left(k_{in}\right)\right]\right\}}^{2}+{\left\{C^{-1}\left[C_{k}\left(k_{out}\right)\right]\right\}}^{2}\right)}{2\left(1-{\hat{\rho }}^{2}\right)}\right] \end{array}$$

(28) $$\begin{array}{l} =\frac{p\left(k_{in}\right)p\left(k_{out}\right)}{\sqrt{1-{\hat{\rho }}^{2}}}\exp\left\{\frac{\hat{\rho }C^{-1}\left[C_{k}\left(k_{in}\right)\right]C^{-1}\left[C_{k}\left(k_{out}\right)\right]}{1-{\hat{\rho }}^{2}}\right\}\\ \times \exp\left[\frac{-{\hat{\rho }}^{2}\left({\left\{C^{-1}\left[C_{k}\left(k_{in}\right)\right]\right\}}^{2}+{\left\{C^{-1}\left[C_{k}\left(k_{out}\right)\right]\right\}}^{2}\right)}{2\left(1-{\hat{\rho }}^{2}\right)}\right] \end{array}$$

**Figure 1:**
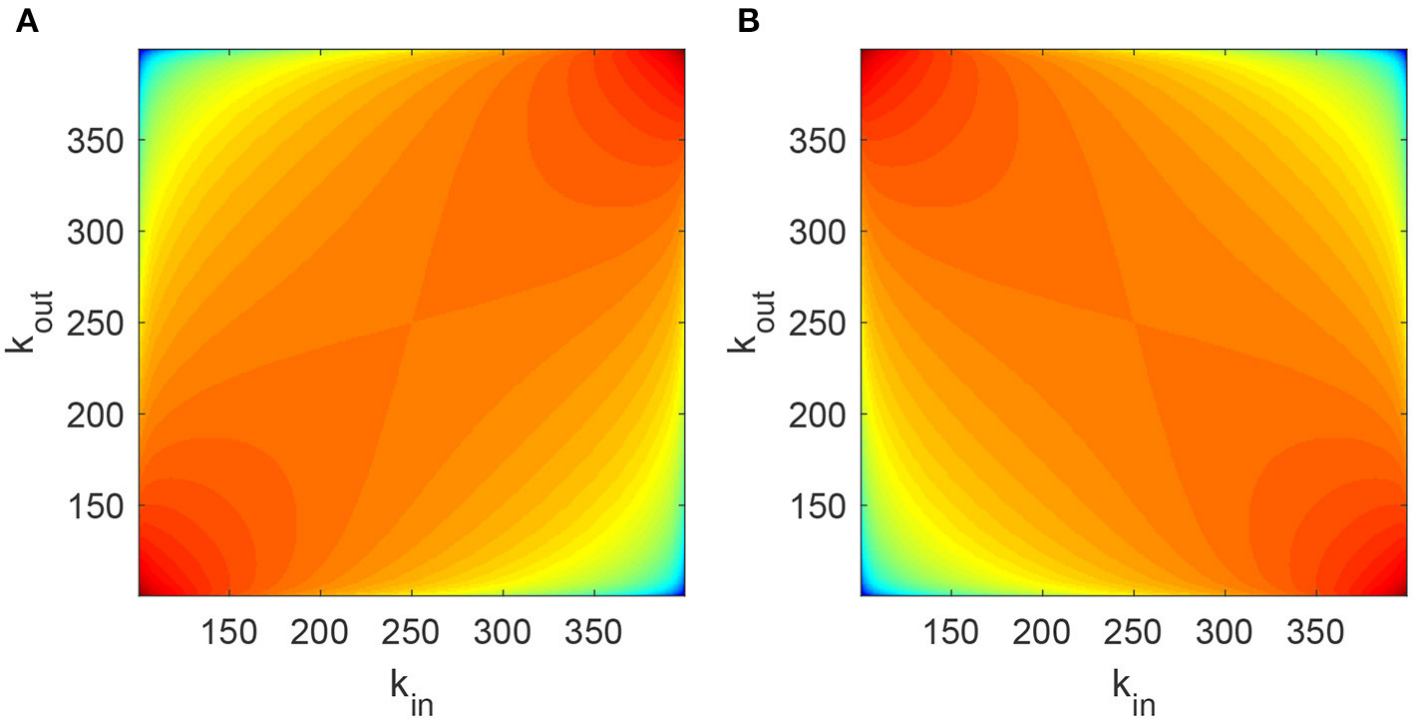
Joint degree distribution $P\left(k_{in},k_{out},\hat{\rho }\right)$ for **(A)** $\hat{\rho }=0.5$ and **(B)** $\hat{\rho }=-0.5$. The log of *P* is shown, with red corresponding to higher values and blue to lower. Parameters: *m* = 100, *M* = 400.

Note the special case *P*(*k*_*in*_, *k*_*out*_, 0) = *p*(*k*_*in*_)*p*(*k*_*out*_), as expected. Several plots of this function are shown in Figure 1.

**Figure 2:**
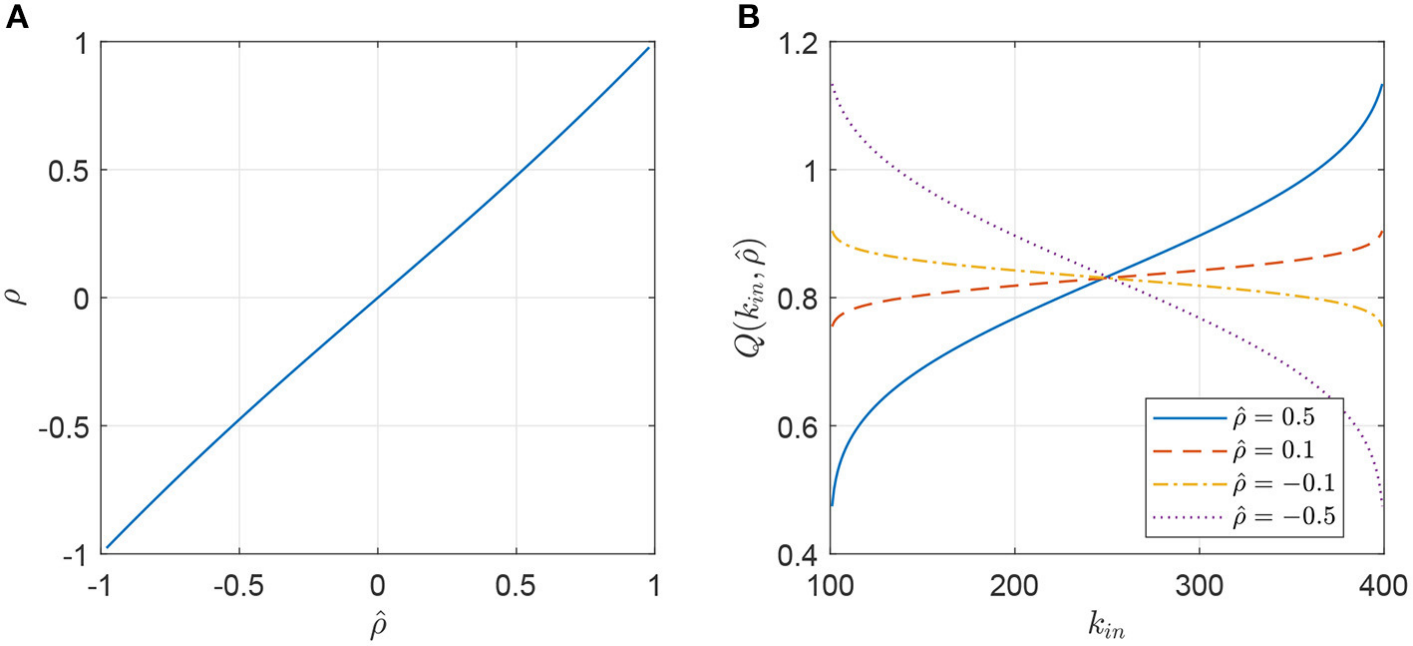
**(A)** Correlation coefficient between the in- and out-degrees of an oscillator, ρ, as a function of the parameter $\hat{\rho }$ used in the Gaussian copula. **(B)** The function $Q\left(k_{in},\hat{\rho }\right)$ (Equation 18) for different values of $\hat{\rho }$. Parameters: *m* = 100, *M* = 400.

We also need to relate the parameter $\hat{\rho }$ to ρ, the Pearson correlation coefficient between the in- and out-degrees of a neuron. We have

(29) $$\begin{array}{l} \rho =\frac{\tilde{\Sigma }P\left(k_{in},k_{out},\;\hat{\rho }\right)\left(k_{in}-\langle k\rangle \right)\left(k_{out}-\langle k\rangle \right)}{\sqrt{\tilde{\Sigma }P\left(k_{in},k_{out},\hat{\rho }\right){\left(k_{in}-\langle k\rangle \right)}^{2}}\sqrt{\tilde{\Sigma }P\left(k_{in},k_{out},\hat{\rho }\right){\left(k_{out}-\langle k\rangle \right)}^{2}}} \end{array}$$

where $\tilde{\Sigma }$ indicates a sum over all *k*_*in*_ and *k*_*out*_. This relationship is numerically determined and shown in Figure 2A, and it is nearly the identity. Note that the sums in (29) are over *m* + 1 ≤ *k* ≤ *M* − 1, since $P\left(k_{in},k_{out},\hat{\rho }\right)$ is undefined for *k* = *m, M*.

We can also calculate the function $Q\left(k_{in},\hat{\rho }\right)$ (Equation 18) where $P\left(k_{in},k_{out},\hat{\rho }\right)$ is given in (28). This function is shown in Figure 2B, where we see that increasing $\hat{\rho }$ gives more weight to high in-degree nodes and less to low in-degree nodes and vice versa. This can be understood by realizing that $Q\left(k_{in},\hat{\rho }\right)$ is the “weight” given to outputs from oscillators with in-degree *k*_*in*_. If, for example, $\hat{\rho }>0$, then oscillators with high in-degree will be likely to have high out–degree, and thus their output should be weighted more.

### 3.2. Results

**Figure 3:**
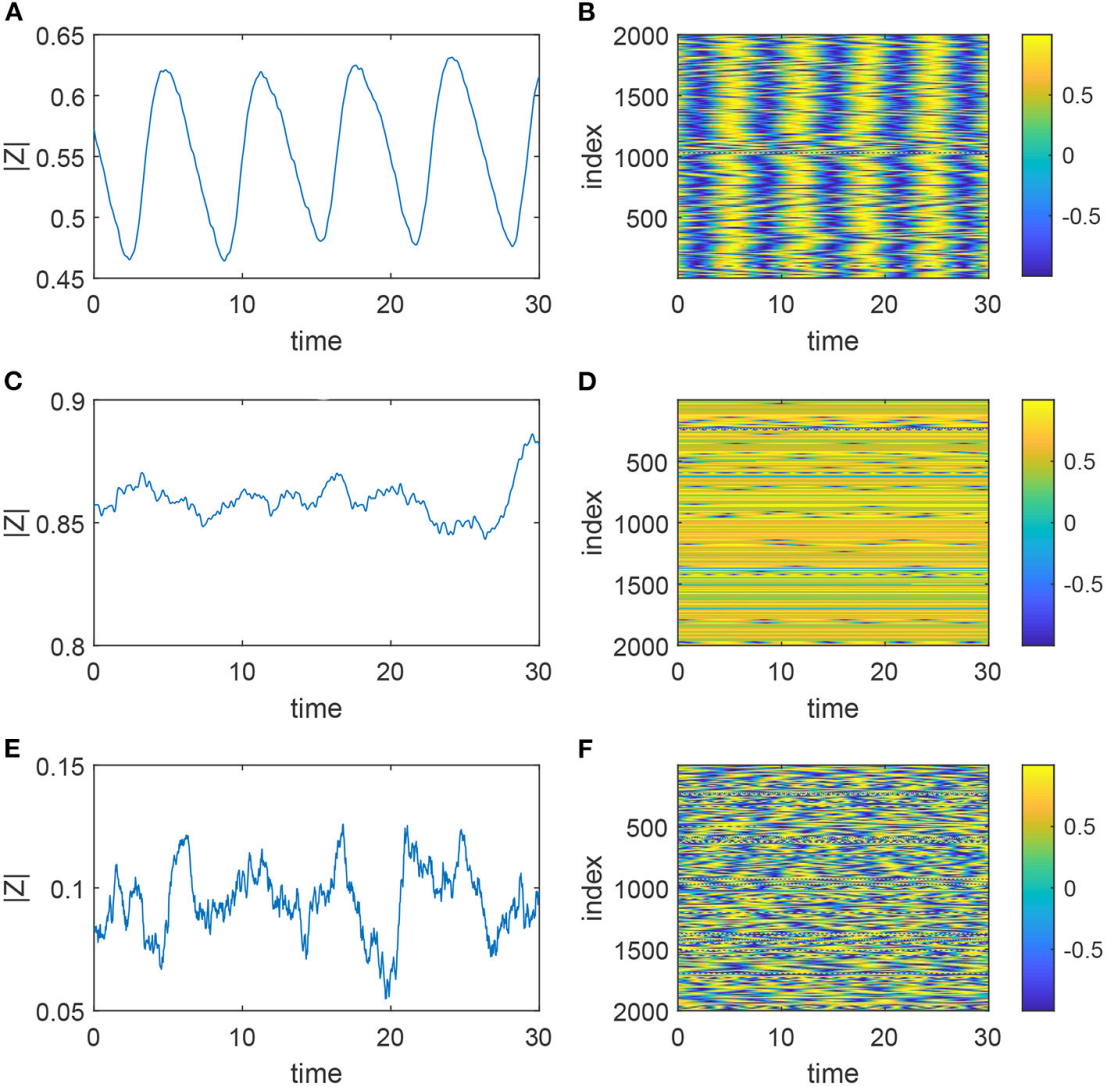
Dynamics of the system (1) with uncorrelated degrees. **(A,B)** Correspond to (ϵ, Δ) = (0.2, 0.05) (synchronous state), **(C,D)** to (ϵ, Δ) = (0.8, 0.05), and **(E,F)** to (ϵ, Δ) = (0.2, 0.5) (asynchronous states). The left panels show the magnitude of the global order parameter, and the right show sin θ_*j*_. Other parameters: ω_0_ = 1, β = 0, *m* = 100, *M* = 400, *N* = 2000.

We set *q* = 4 (so *a*_*q*_ = 8/35 and *C*_0_ = 35/8, *C*_1_ = 7/2, *C*_2_ = 7/4, *C*_3_ = 1/2, *C*_4_ = 1/16), ω_0_ = 1, and consider four different values of β: 0, 0.5, 0.7, and 1 (all corresponding to type-II oscillators). There are two types of behavior typically seen in such a network: synchronous and asynchronous (Pazó and Montbrió, 2014), although the fraction of oscillators actually oscillating can vary in the asynchronous states. Increasing ϵ (the strength of coupling) tends to destroy synchronous behavior through a saddle-node-on-invariant-circle (SNIC) bifurcation, as many of the oscillators “lock” at an approximate fixed point. Increasing Δ (the spread of intrinsic frequencies) tends to destroy synchronous behavior through a Hopf bifurcation, as the oscillators become too dissimilar to synchronize (Pazó and Montbrió, 2014). Examples of typical behavior in a default network are shown in Figure 3. The global order parameter for a network of *N* phase oscillators is a measure of their synchrony, and is defined as (Strogatz, 2000)

(30) $$\begin{array}{l} Z=\frac{1}{N}\overset{N}{\underset{j=1}{\sum }}e^{i\theta_{j}}. \end{array}$$

We see that its magnitude has large, nearly periodic oscillations in the synchronous state, but is approximately constant in the asynchronous state—note the different vertical scales in Figures 3A,C,E. Note as well the high |*Z*| value reflects a large fraction of quiescent oscillators in Figures 3C,D—a “trivial synchrony.”

**Figure 4:**
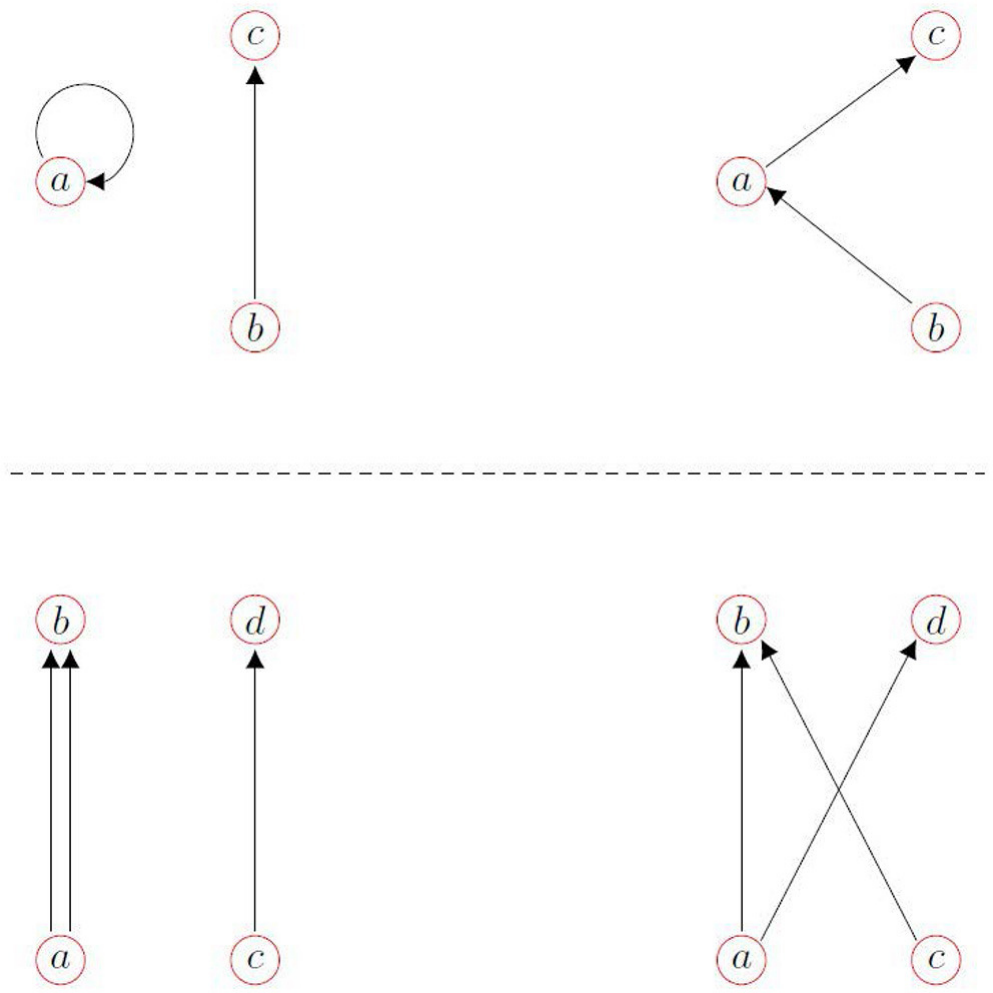
(Top) We remove a self-connection to oscillator *a* (left) by rewiring the randomly chosen connection from oscillator *b* to *c*, giving the configuration at the right. (Bottom) we remove the double connection from oscillator *a* to *b* by rewiring the randomly chosen connection from oscillator *c* to *d*, giving the configuration at the right.

The network whose behavior is shown in Figure 3 was created using the configuration model (Newman, 2003). Such a network typically has both self-connections (i.e., an oscillator is connected to itself) and multiple connections from one particular oscillator to another. We remove these in a random way as shown in Figure 4. For a self-connection we randomly choose another connection and reconnect as in the top panel. For a double connection we randomly choose another connection and reconnect as in the bottom panel.

**Figure 5:**
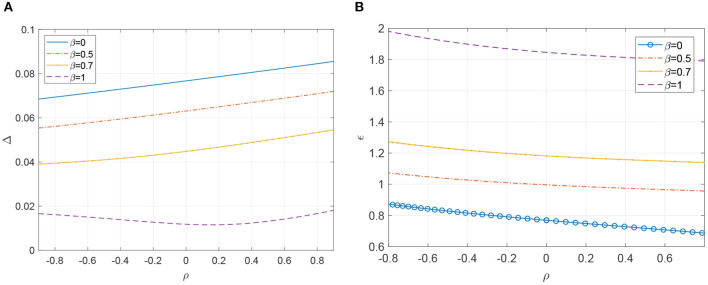
**(A)** Hopf bifurcation curves of the fixed point of (19). A stable periodic orbit exists below the curve. Other parameters: ϵ = 0.2, ω_0_ = 1, *m* = 100, *M* = 400. **(B)** SNIC bifurcation curve (β = 0, blue circles joined by line) and Hopf bifurcation curves (β = 0.5, 0.7 and 1) for (19). For each value of β a stable periodic orbit exists below the curve. Other parameters: Δ = 0.05, ω_0_ = 1, *m* = 100, *M* = 400.

We now investigate the effects of varying $\hat{\rho }$ and thus ρ on the dynamics of Equation (19). As mentioned, it is known that increasing Δ (making the intrinsic frequencies more diverse) destroys the synchronous state in a supercritical Hopf bifurcation (Pazó and Montbrió, 2014). In Figure 5A, we show how the value of Δ at which this bifurcation occurs varies as a function of ρ, for four different values of β. We vary $\hat{\rho }$ but interpolate the data shown in Figure 2A in order to plot the curves in Figure 5A as functions of ρ. We see that increasing ρ increases the value of Δ at which the bifurcation occurs, at least for small β, and vice versa, but the effect is small compared with that of varying β. Put another way, for a fixed value of Δ, increasing ρ can cause macroscopic oscillations within the network (at least for β close to zero).

We now fix Δ = 0.05 and consider the effects of varying both ρ and ϵ (the strength of coupling between oscillators). It is known that for an all-to-all coupled network increasing ϵ destroys the synchronous state in a SNIC bifurcation (Pazó and Montbrió, 2014). For our network this is also what happens for β = 0, as shown in Figure 5B (blue circles joined by line). However, for β = 0.5, 0.7, and 1, there is instead a supercritical Hopf bifurcation as ϵ increases, in contrast with the situation for all-to-all coupled network (for these values of ω_0_, β and Δ), illustrating a nontrivial effect of network structure: even the type of bifurcation occurring is changed. These curves of Hopf bifurcations are also shown in Figure 5B and we see that increasing ρ decreases the value of ϵ at which the synchronous solution is destroyed and vice versa. Note that between β = 0 and β = 0.5, guided by the results for the fully-connected network (Pazó and Montbrió, 2014), we expect there to be several curves of Hopf, homoclinic, and saddle-node bifurcations in Figure 5B organized around a Takens-Bogdanov and a saddle-node separatrix-loop point (Gallego et al., 2017), but we have not investigated them here. The results in Figure 5 have been compared with those from simulation of the full network (Equation 1) and found to agree very well (results not shown).

## 4. Between Neuron Degree Correlations

We now turn to the question of correlations between connected oscillators based on their degrees, often referred to as degree assortativity (Foster et al., 2010; Bläsche et al., 2020). Assortativity has often been studied in undirected networks, where a node simply has a degree, rather than in- and out-degrees (Restrepo and Ott, 2014). Here we consider directed networks, which a small number of previous authors have considered (De Franciscis et al., 2011; Avalos-Gaytan et al., 2012; Schmeltzer et al., 2015; Kähne et al., 2017), although they have often imposed other structure on the network such as equal in- and out-degrees for each model neuron.

**Figure 6:**
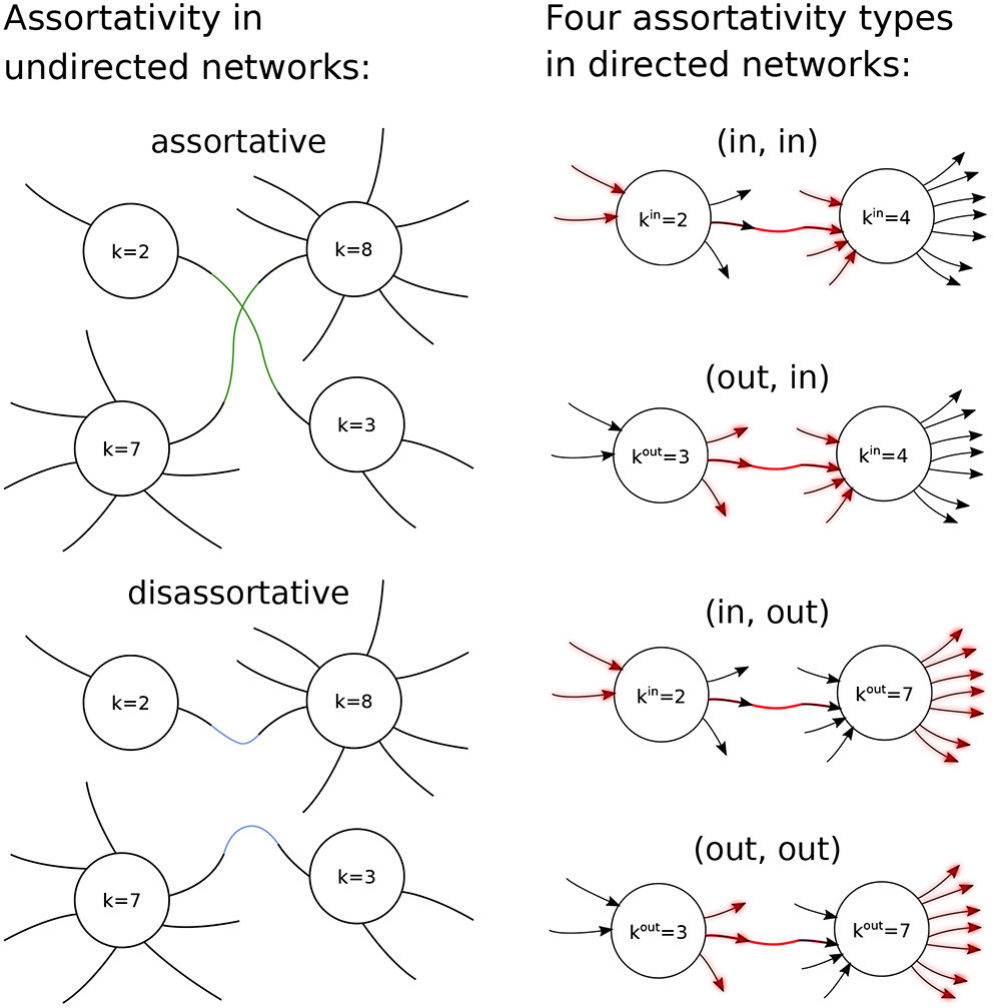
Assortativity in undirected and directed networks. An undirected network (left) is assortative if high degree nodes are more likely to be connected to high degree nodes, and low to low, than by chance (top left). Such a network is disassortative if the opposite occurs (bottom left). (Here, the degree of a node is given by *k*.) In directed networks (right) there are four possible kinds of assortativity. The probability of a connection (red) depends on the number of red shaded links of the sending (left) and receiving (right) node. (Here, either the in-degree, *k*^*in*^, or out-degree, *k*^*out*^, of a node is the relevant quantity).

Because we consider directed networks there are four possible types of degree assortativity, between either the in- or out-degree of the “upstream” (sending) oscillator and either the in- or out-degree of the “downstream” (receiving) oscillator (see Figure 6).

Roughly speaking, degree assortativity can be thought of in this way: given an upstream oscillator with specific in- and out-degrees, and a downstream oscillator with specific in- and out-degrees, one can calculate the probability of a connection from the upstream to the downstream oscillator. If this probability—averaged over the network—is other than that expected by chance, and is further dependent on the degrees of the oscillators, the network shows degree assortativity. One can use this idea to create networks with assortativity, by creating connections where they would typically not occur.

A measure of assortativity for a network with a given connectivity matrix *A* is by way of calculating the four Pearson correlation coefficients *r*(α, γ) with α, γ ∈ [in, out] given by

(31) r(α, γ)=∑e=1Ne(ukea−〈kαu〉)(dkeγ−〈kγd〉)∑e=1Ne(ukea−〈kαu〉)2 ∑e=1Ne(dkeγ−〈kγd〉)2

where

(32) 〈kuα〉=1Ne∑e=1Neukeα   and   〈kdγ〉=1Ne∑e=1Nedkeγ,

*N*_*e*_ being the number of edges and the leading superscript *u* or *d* refers to the “upstream” or “downstream” oscillator of the respective edge (Bläsche et al., 2020). For example the upstream node's in-degree of the second edge would be k2inu. Note that there are four mean values to compute.

To induce assortativity within a network we start by randomly choosing in-degrees and out-degrees from the distribution given in Equation (20). If the total number of out-degrees does not equal that of the in-degrees (i.e., the network cannot be created; Anstee, 1982) we choose again until it does. We then use the configuration model (Newman, 2003) with these prescribed degrees to create the network, and utilize the same procedure as described earlier for removal of self- and multiple-connections (see Figure 4).

To induce assortativity of the form (α, γ) we randomly choose two edges, one connecting oscillator *j* to oscillator *i* and another connecting oscillator *l* to oscillator *h*. We calculate their contribution to the numerator of (31)

(33) c∥=(kja−〈kau〉)(kiγ−〈kγd〉)+(klα−〈kau〉)(khγ−〈kγd〉)

and the contribution if we replaced these two edges with one connecting oscillator *j* to oscillator *h* and another connecting oscillator *l* to oscillator *i*:


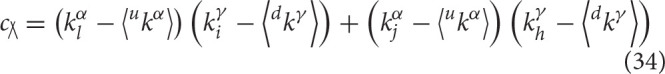


If 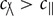 we make the swap, otherwise we do not. We then repeat this process many times, storing *A*, and calculating the value of *r*(α, γ) at regular intervals.

We now discuss how to implement the system Equation (11). Choosing *m* = 100, *M* = 400, *k*_*in*_, and *k*_*out*_ take on values in {100, 101, 102, …400} and thus there are 301 × 301 possible values of **k**. Considering that we use a network of size *N* = 2, 000 it is clear that there may be many values of **k** for which there is not even one oscillator in the network. Thus, we coarse-grain by degree: we divide the interval [100, 400] into 15 equal-size bins with centers ${\hat{k}}_{in,1},{\hat{k}}_{in,2},\ldots ,{\hat{k}}_{in,15}$ and describe the state of an oscillator by the value of *b* associated with the 2D bin it is in (there are 15 × 15 of these 2D bins). We can think of Equation (11) as being a matrix-valued ODE, with the (*i, j*)th element of the matrix being $b\left({\hat{k}}_{out,i},{\hat{k}}_{in,j},t\right)$. We can easily convert this to a vector-valued ODE by stacking the columns of $b\left({\hat{k}}_{out},{\hat{k}}_{in},t\right)$, from left to right, into a vector, $\hat{b}\left(t\right)$, where the *s*th entry is ${\hat{b}}_{s}\left(t\right)=b\left({\hat{k}}_{out,i},{\hat{k}}_{in,j},t\right)$ and *s* = *i* + 15(*j* − 1). Note that *i, j* ∈ {1, 2, …15} and *s* ∈ {1, 2, …225}.

Dropping the hat on *b* we have

(35) $$\begin{array}{l} \frac{db_{s}\left(t\right)}{dt}=\frac{\epsilon e^{-i\beta }R_{s}\left(t\right)}{2}+\left[i\omega_{0}-\Delta +i\epsilon \sin\beta R_{s}\left(t\right)\right]b_{s}\left(t\right)\\ \;-\frac{\epsilon e^{i\beta }R_{s}\left(t\right)}{2}{\left[b_{s}\left(t\right)\right]}^{2} \end{array}$$

for *s* ∈ {1, 2, …225} where we define

(36) $$\begin{array}{l} G_{s}\left(t\right)=a_{n}\left[C_{0}+\overset{n}{\underset{j=1}{\sum }}C_{j}\left\{{\left[b_{s}\left(t\right)\right]}^{j}+{\left[{\bar{b}}_{s}\left(t\right)\right]}^{j}\right\}\right] \end{array}$$

We need to calculate *R*_*s*_(*t*) from *G*_*s*_(*t*) using the equivalent of (8). We can write the analog of (8) as

(37) $$\begin{array}{l} R_{s}\left(t\right)=\frac{1}{\langle k\rangle }\overset{225}{\underset{s^{\prime }=1}{\sum }}E\left(s,s^{\prime }\right)G_{s^{\prime }}\left(t\right) \end{array}$$

where *E*(*s, s*′) encodes the connectivity from the 2D bin with index *s*′ to that with index *s*. Given the connectivity matrix *A* it is straightforward to calculate *E*(*s, s*′) as explained in Bläsche et al. (2020). *E* can be thought of as a 225 × 225 matrix, with (*i, j*)th entry *E*(*i, j*), so we can write Equation (37) as

(38) $$\begin{array}{l} R\left(t\right)=\frac{1}{\langle k\rangle }EG\left(t\right) \end{array}$$

where *R* and *G* are vector-valued variables and Equation (35) is just the *s*th component of a vector-valued ODE.

Since we have recorded *A* at discrete values of the correlation coefficient *r*, we can also calculate *E* at these values. To form a parameterized family, *E*(*r*), we fit a quadratic to each entry of *E* as a function of *r*, i.e., we write $E_{ij}\left(r\right)=B_{ij}r^{2}+C_{ij}r+D_{ij}$ for *i, j* ∈ [1, 225], using linear least-squares. We can then efficiently evaluate an approximation of *E*(*r*) as

(39) $$\begin{array}{l} E\left(r\right)=Br^{2}+Cr+D \end{array}$$

where the (*i, j*)th entry of *B* is *B*_*ij*_ etc. In summary, we have a parameterized set of ODEs, where *r* is one of the parameters. Note that we only vary one of the four *r*(α, γ) at a time.

### 4.1. Results

**Figure 7:**
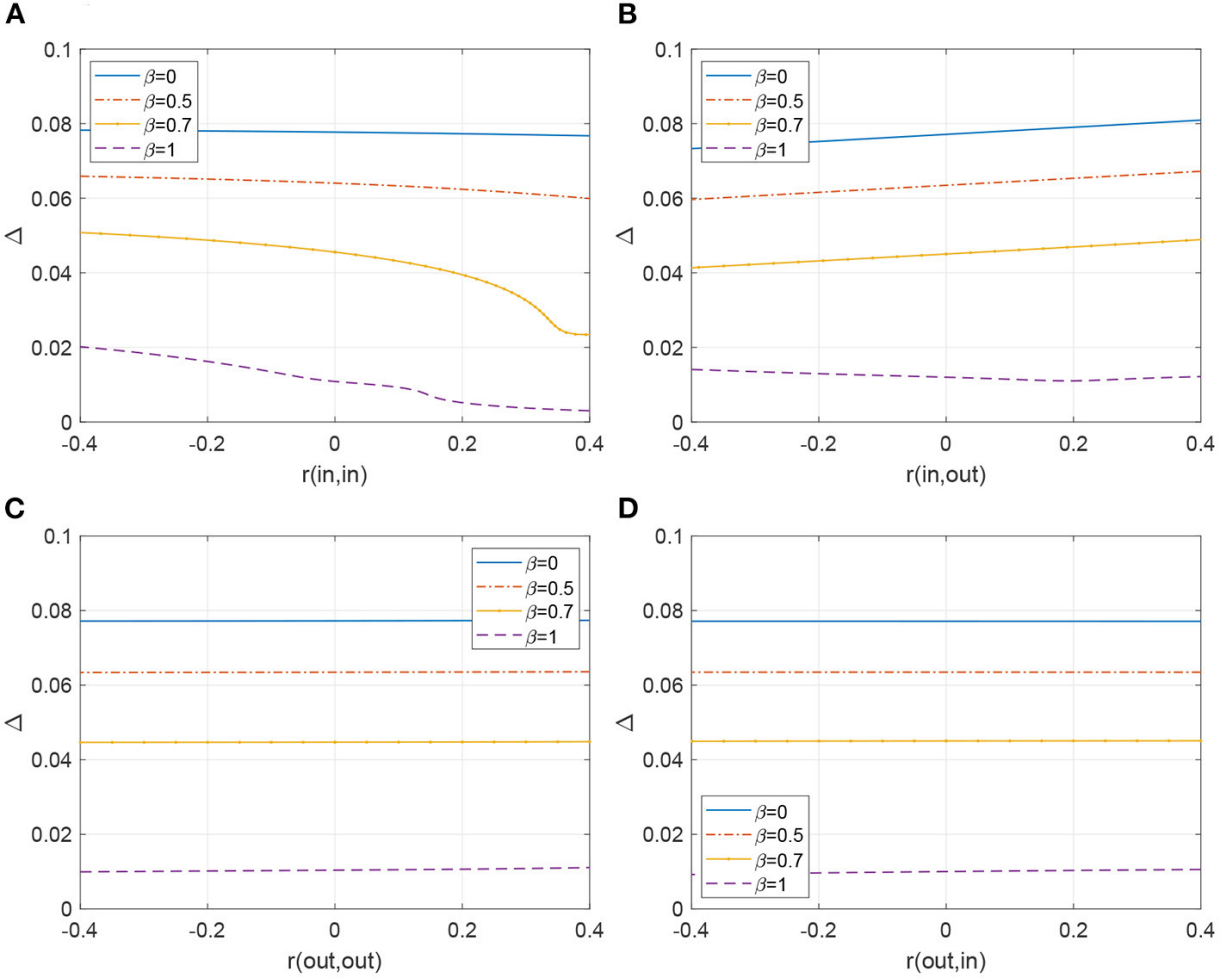
**(A–D)** Hopf bifurcation curves as both Δ and one of the types of assortativity are varied. A stable periodic orbit exists below the curve. Other parameters: ϵ = 0.2, ω_0_ = 1, *m* = 100, *M* = 400.

**Figure 8:**
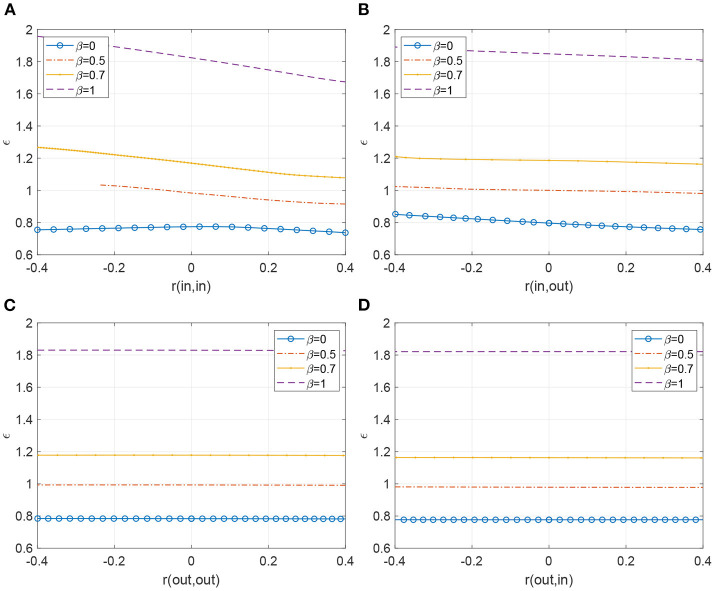
**(A–D)** SNIC bifurcation curve (β = 0, blue circles joined by line) and Hopf bifurcation curves (β = 0.5, 0.7 and 1) as both ϵ and one of the types of assortativity are varied. For each value of β a stable periodic orbit exists below the curve. Other parameters: Δ = 0.05, ω_0_ = 1, *m* = 100, *M* = 400. In the top-left panel, for β = 0.5 the curve terminates as *r* is decreased.

The results are shown in Figure 7, where we vary Δ and the four *r*(α, γ) for four different values of β, and Figure 8, where we vary ϵ and the four *r*(α, γ) for the same four values of β. As was seen in Bläsche et al. (2020), assortativities of the type r(out,out) and r(out,in) have no discernable effect on the bifurcations, whereas the other two types do. We can understand this by realizing that the dynamics of an oscillator depend only on its inputs. Since an oscillator's dynamics are independent of its downstream oscillators, neither the r(out,out) nor the r(out,in) assortativities influence the overall network dynamics as shown in all the traces of Figures 7C,D. Note, this dynamic interplay is quite different for a network with strong preferential attachment between high in-degree and high out-degree oscillators as when r(in,out) is positive (Figure 7B). The influence of the upstream oscillator (with high in-degree, receiving multiple inputs) is amplified or “passed on” to more oscillators via its downstream companion with high out-degree. This pair with high input and high output is thus far more influential than, say, a pair of oscillators preferentially attached according to the upstream node's out-degree. In that scenario, the upstream node of an attached pair may only integrate a small number of inputs (low in-degree), whose behavior is strikingly distinct from an oscillator with many inputs (high in-degree).

Analogously, a positive r(in,in) assortativity demonstrates preferential attachment between high in-degree upstream and downstream pairs of oscillators. In this case, they are relatively potent integrators and concentrators of upstream impulses. We see in Figure 7A, the influence of high r(in,in) where the parameter space in which stable periodic orbits exist shrinks, increasing sensitivity to the destructive influence of Δ on synchrony.

## 5. Correlated Heterogeneity

We have so far assumed that the parameters ω_0_ and Δ (the mean and width, respectively, of the distribution of intrinsic frequencies, see Equation 10) and β (the parameter in the phase response curve, see Equation 2) are the same for each oscillator, but now consider the case of them being correlated with a structural property of an oscillator such as its in-degree or out-degree. Correlating an oscillator's frequency with its degree is known to cause “explosive” synchronization in undirected networks of coupled phase oscillators, for example Liu et al. (2013), Gómez-Gardeñes et al. (2011), and Boccaletti et al. (2016), and we are interested in whether similar effects occur in networks of Winfree oscillators. For simplicity we will use linear relationships between a parameter and its relevant degree.

### 5.1. In-Degree

We first consider the case of correlation with in-degree. Assuming neutral assortativity and independence between an oscillator's in- and out-degree, following the reasoning in section 3 the dynamics of *b* depend only on in-degree and are governed by

$$\begin{array}{l} \frac{\partial b\left(k_{in}\right)}{\partial t}=\frac{\epsilon e^{-i\beta }R\left(k_{in}\right)}{2}+\left[i\omega_{0}-\Delta +i\epsilon \sin\beta R\left(k_{in}\right)\right]b\left(k_{in}\right)\\ \;-\frac{\epsilon e^{i\beta }R\left(k_{in}\right)}{2}{\left[b\left(k_{in}\right)\right]}^{2} \end{array}$$

for *k*_*in*_ = *m, m* + 1, …*M* where

(41) $$\begin{array}{l} R\left(k_{in},t\right)=\frac{k_{in}}{{\langle k\rangle }^{2}}\underset{k_{in}^{\prime }}{\sum }\underset{k_{out}^{\prime }}{\sum }p\left(k_{in}^{\prime }\right)p\left(k_{out}^{\prime }\right)k_{out}^{\prime }G\left(k_{in}^{\prime },t\right)\\ \;=\frac{k_{in}}{\langle k\rangle }\underset{k_{in}^{\prime }}{\sum }p\left(k_{in}^{\prime }\right)G\left(k_{in}^{\prime },t\right) \end{array}$$

where *G* is given by (12) but with the degree dependence being on only *k*_*in*_.

**Figure 9:**
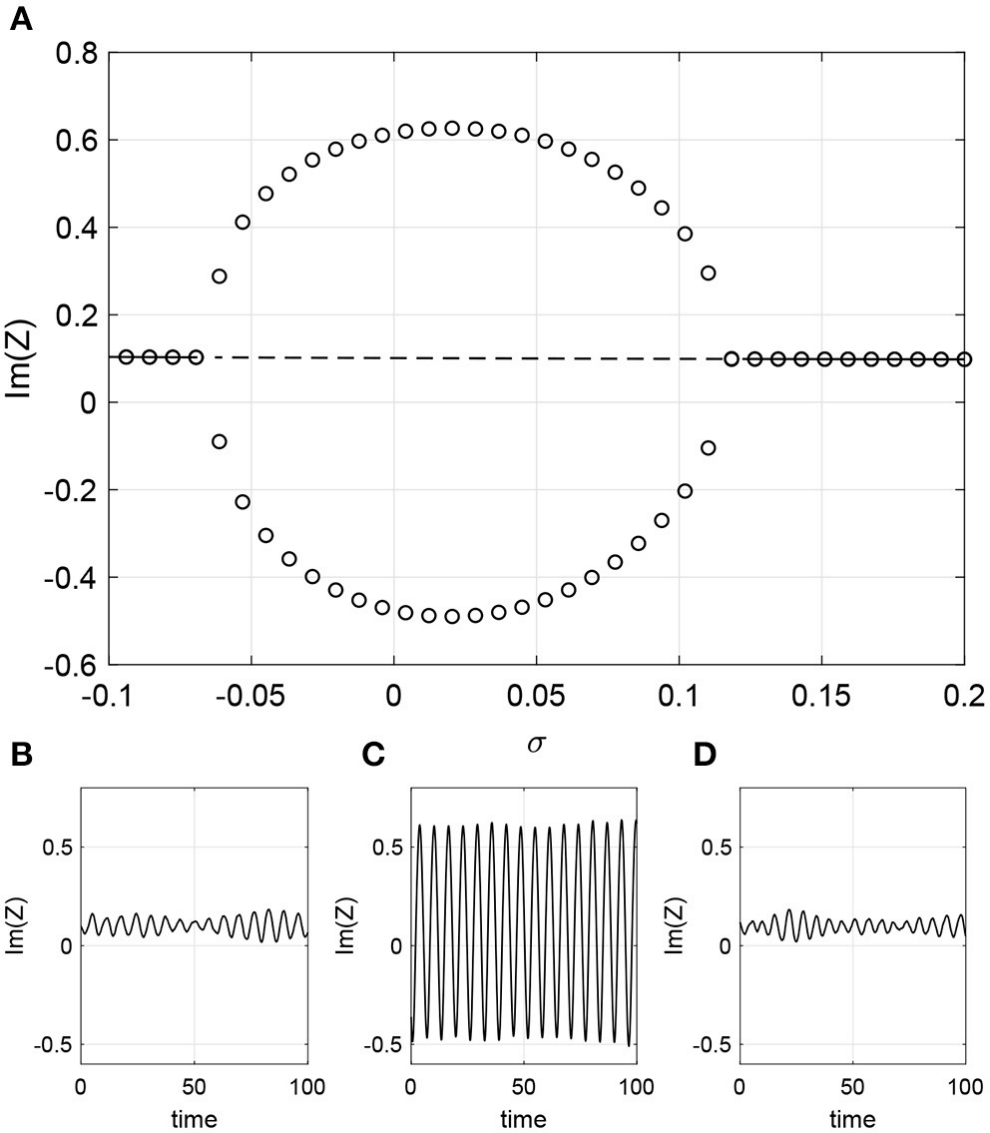
Intrinsic frequency dependence $\omega_{0}\left(k_{in}\right)=1+\sigma {\hat{k}}_{in}$. **(A)** Solid line: stable fixed point of Equation (40); dashed line: unstable fixed point. Circles: maximum and minimum over one period of Im(*Z*). Im(*Z*) calculated using (30) for **(B)** σ = −0.1, **(C)** σ = 0, and **(D)** σ = 0.2. Other parameters: Δ = 0.05, β = 0, ϵ = 0.2, *m* = 100, *M* = 400, *N* = 2, 000.

We define a scaled in-degree

(42) $$\begin{array}{l} {\hat{k}}_{in}=2\left(\frac{k_{in}-m}{M-m}\right)-1 \end{array}$$

which varies linearly from −1 to 1 as *k*_*in*_ goes from *m* to *M*, respectively. We first consider the case where ω_0_ is a function of *k*_*in*_. We write $\omega_{0}\left(k_{in}\right)=1+\sigma {\hat{k}}_{in}$ where σ controls the strength of dependence between *k*_*in*_ and ω_0_. (Recall that we previously set ω_0_ = 1.) Setting β = 0, ϵ = 0.2, and Δ = 0.05 we find that when σ = 0 the network is attracted to a stable periodic orbit. However, increasing or decreasing σ causes the oscillations to cease through a Hopf bifurcation as shown in Figure 9A. To visualize the oscillations we define the complex order parameter for (40) as

(43) $$\begin{array}{l} Z\left(t\right)=\frac{1}{M-m+1}\overset{M}{\underset{k_{in}=m}{\sum }}b\left(k_{in},t\right). \end{array}$$

This is an appropriate definition since the distribution of in-degrees is uniform; if it were not we would have to weight the contributions from different *k*_*in*_ values. Figure 9A shows the maximum and minimum over one period of Im(*Z*) for oscillatory solutions, and just Im(*Z*) for fixed points. Simulations of a finite network are shown in the lower panels of Figure 9 (transients not shown) which confirm the results in Figure 9A. The small amplitude oscillations seen in Figures 9A,C are a result of finite size fluctuations about the fixed point of Equation (40), the linearization about which has complex eigenvalues. The amplitude of these oscillations decreases as the number of oscillators used increases (not shown).

One might think that having ω_0_ depend on in-degree broadens the distribution of intrinsic frequencies in the network, which is equivalent in some sense to increasing Δ. However, it is not completely equivalent for several reasons. Firstly, the distribution of all intrinsic frequencies is no longer Lorentzian (although for each oscillator we choose the frequency from a Lorentzian), and depends on both the form of dependence of ω_0_ on *k*_*in*_ (linear in this case) and the distribution of the *k*_*in*_ (uniform in this case). Secondly, the intrinsic frequency of each oscillator now depends on a structural property: its in-degree. But for comparison, the oscillations seen in Figure 9 for σ = 0 are destroyed in a Hopf bifurcation as Δ is increased through ~0.085 (not shown).

**Figure 10:**
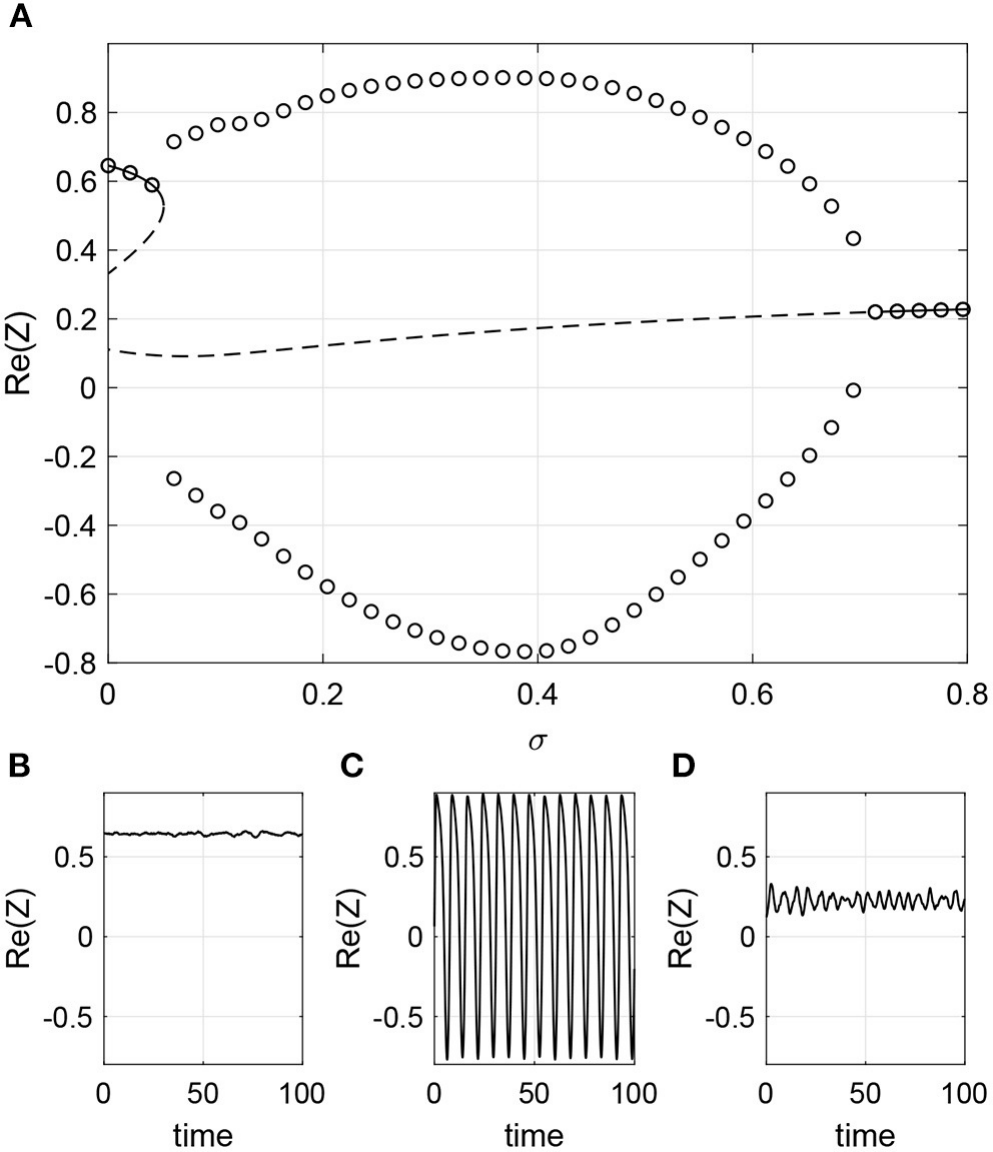
Phase dependency $\beta \left(k_{in}\right)=\sigma \left({\hat{k}}_{in}+1\right)$. **(A)** Solid line: stable fixed point of Equation (40); dashed line: unstable fixed point. Circles: maximum and minimum over one period of Re(*Z*). Re(*Z*) calculated using (30) for **(B)** σ = 0, **(C)** σ = 0.4, and **(D)** σ = 0.8. Other parameters: Δ = 0.05, ω_0_ = 1, ϵ = 0.8, *m* = 100, *M* = 400, *N* = 2, 000.

Next consider β being a function of *k*_*in*_. In order to not have negative β we set $\beta =\sigma \left({\hat{k}}_{in}+1\right)$. We choose ω_0_ = 1, ϵ = 0.8, Δ = 0.05. For these parameters the network is attracted to a stable fixed point. However, increasing σ first induces oscillations through a SNIC bifurcation and then destroys them through a Hopf bifurcation, as shown in Figure 10A. Simulations of a finite network are shown in the lower panels of Figure 10 and these are consistent with the results in Figure 10A.

**Figure 11:**
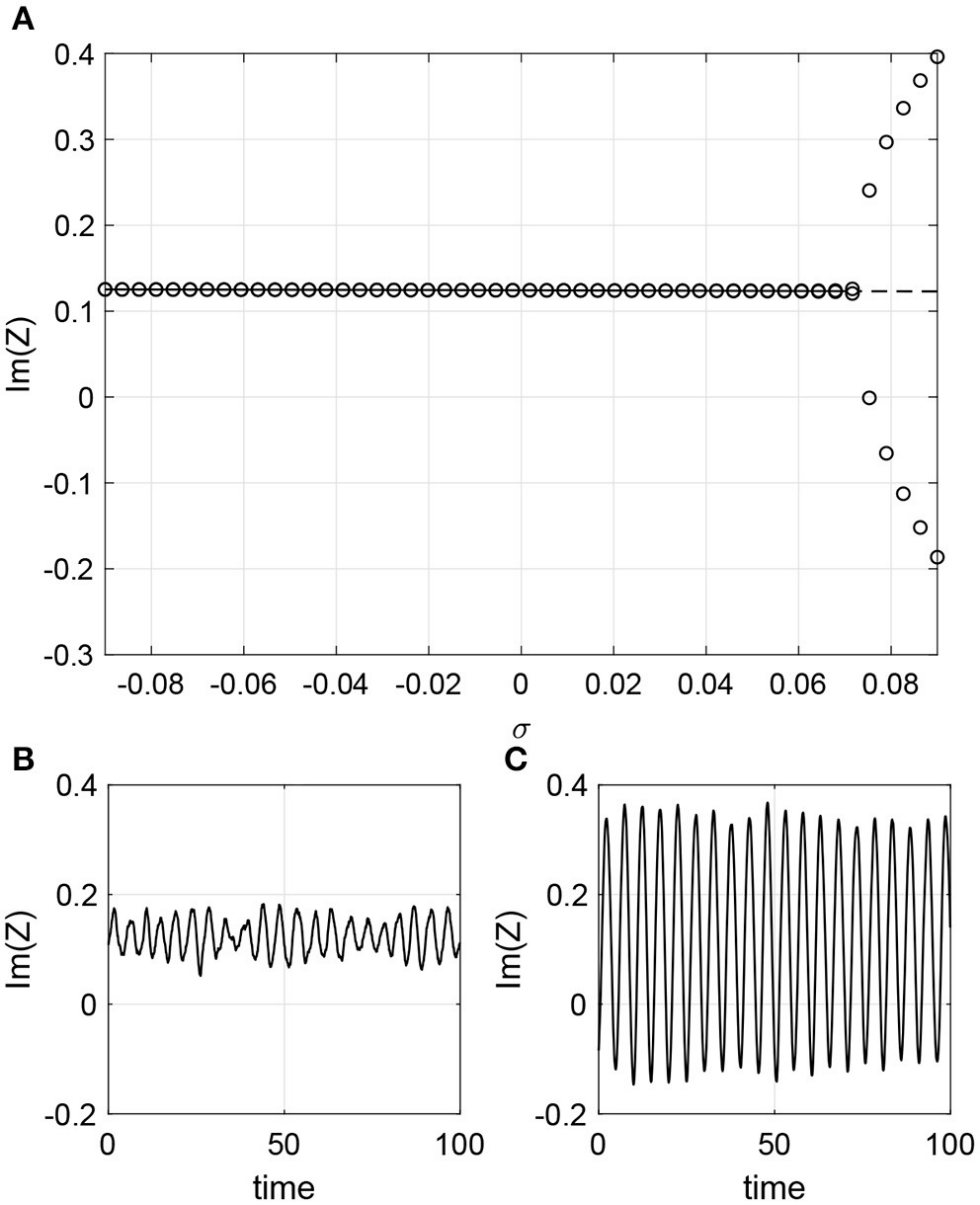
Heterogeneity dependency $\Delta =0.09+\sigma {\hat{k}}_{in}$. **(A)** Solid line: stable fixed point of (40); dashed line: unstable fixed point. Circles: maximum and minimum over one period of Im(*Z*). Im(*Z*) calculated using (30) for **(B)** σ = 0 and **(C)** σ = 0.09. Other parameters: β = 1, ω_0_ = 1, ϵ = 0.6, *m* = 100, *M* = 400, *N* = 2, 000.

As a third possibility we let Δ depend on *k*_*in*_. Δ (the width of the distribution of intrinsic frequencies) cannot be negative so we set $\Delta =0.09+\sigma {\hat{k}}_{in}$ and consider only −0.09 ≤ σ ≤ 0.09. We set other parameters ω_0_ = 1, β = 1 and ϵ = 0.6. A Hopf bifurcation occurs as σ is increased as shown in Figure 11A. Simulations of a finite network are shown in the lower panels of Figure 11. Significant oscillations are seen for σ = 0, and the amplitude of oscillations for σ = 0.09 is less than expected. However, we repeated this type of simulation with *N* = 5, 000 and found that the amplitude of oscillations with σ = 0.09 better matched the results in Figure 11A (i.e., were bigger than seen for *N* = 2, 000) and that the amplitude of oscillations for σ = 0 were slightly smaller than seen for *N* = 2, 000 (results not shown), suggesting that this apparent disagreement is a finite-size effect.

### 5.2. Out-Degree

Now consider the possibility that one of ω_0_, Δ, and β are correlated with an oscillator's out-degree. From Equation (11), it is clear that even for neutral assortativity, *b* will depend on both *k*_*in*_ and *k*_*out*_. Thus, the relevant equations are

(44) $$\begin{array}{l} \frac{\partial b}{\partial t}=\frac{\epsilon e^{-i\beta }R}{2}+\left[i\omega_{0}-\Delta +i\epsilon \sin\beta R\right]b-\frac{\epsilon e^{i\beta }R}{2}b^{2} \end{array}$$

where *b* is a function of both *k*_*in*_ and *k*_*out*_, but *R* is a function of *k*_*in*_ only:

(45) $$\begin{array}{l} R\left(k_{in},t\right)=\frac{k_{in}}{{\langle k\rangle }^{2}}\underset{k_{in}^{\prime }}{\sum }\underset{k_{out}^{\prime }}{\sum }p\left(k_{in}^{\prime }\right)p\left(k_{out}^{\prime }\right)k_{out}^{\prime }G\left(k_{in}^{\prime },k_{out}^{\prime },t\right) \end{array}$$

where *G* is given by Equation (12).

#### 5.2.1. Computational Approach

If *J* = *M* − *m* + 1 is the number of distinct in-degrees (and out-degrees) then *b* can be thought of as a *J* × *J* matrix with *J*^2^ entries. This is too many to deal with computationally, so we discretize in degree space. In the same way that one can approximate a definite integral using Gaussian quadrature, it is possible to approximate a double sum like that in (45) using a double sum over far fewer points (Engblom, 2006). The theory is explained in Laing and Bläsche (2020), but put briefly we define an inner product on either degree space

(46) $$\begin{array}{l} \left(f,g\right)=\overset{M}{\underset{k=m}{\sum }}f\left(k\right)g\left(k\right) \end{array}$$

and assume that there is a corresponding set of orthogonal polynomials {*q*_*n*_(*k*)}_0≤*n*_ associated with this product. We choose a positive integer *s* and let {*k*_*i*_}_*i*=1,…*s*_ be the roots of *q*_*s*_, found using the Golub-Welsch algorithm, and {*w*_*i*_} be the weights associated with these roots. The approximation of the double sum in (45) is then

(47) $$\begin{array}{l} \underset{k_{in}^{\prime }}{\sum }\underset{k_{out}^{\prime }}{\sum }p\left(k_{in}^{\prime }\right)p\left(k_{out}^{\prime }\right)k_{out}^{\prime }G\left(k_{in}^{\prime },k_{out}^{\prime },t\right)\approx \overset{s}{\underset{i=1}{\sum }}\overset{s}{\underset{j=1}{\sum }}w_{i}w_{j}k_{j}G\left(k_{i},k_{j},t\right) \end{array}$$

**Figure 12:**
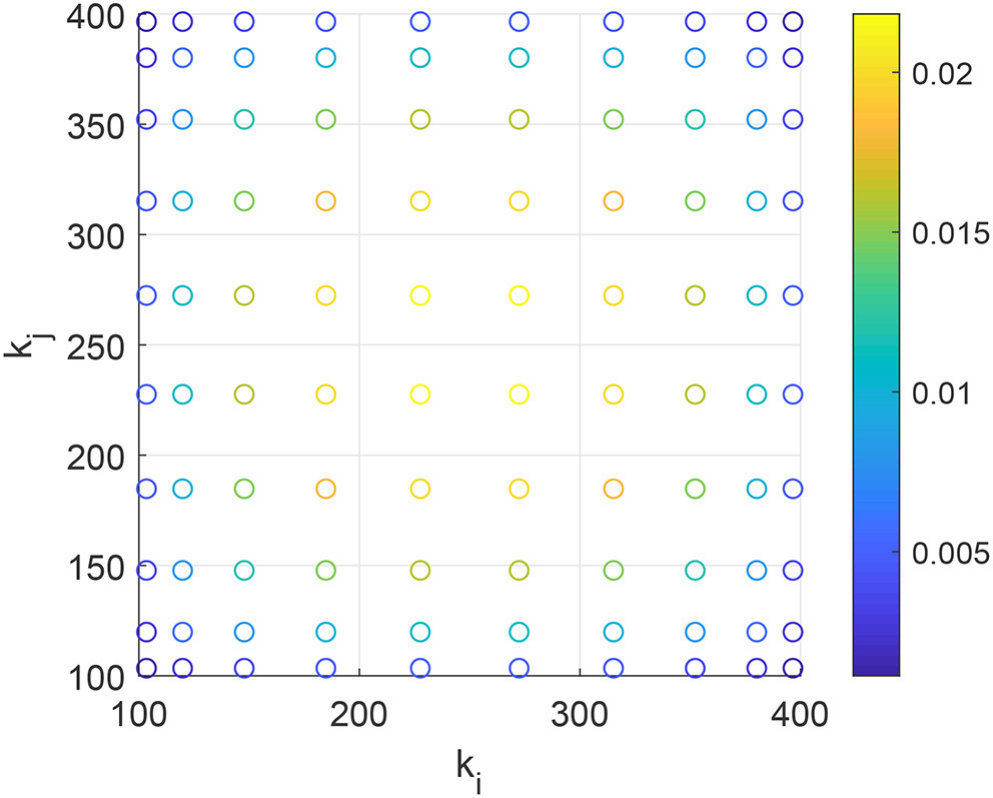
Non-uniform 2D grid of degrees as explained in section 5.2.1 with *m* = 100, *M* = 400, *s* = 10. The color shows the weight, *w*_*i*_*w*_*j*_, associated with each point.

Note that the *k*_*i*_ are not integer-valued. We thus solve (44) on the non-uniform 2D grid of *s*^2^ “virtual” degree. An example for *s* = 10 is shown in Figure 12.

The convergence with *s* is observed to be geometric (not shown) and we use *s* = 20 to calculate the results below.

#### 5.2.2. Results

We write

(48) $$\begin{array}{l} {\hat{k}}_{out}=2\left(\frac{k_{out}\;-\;m}{M\;-\;m}\right)-1. \end{array}$$

**Figure 13:**
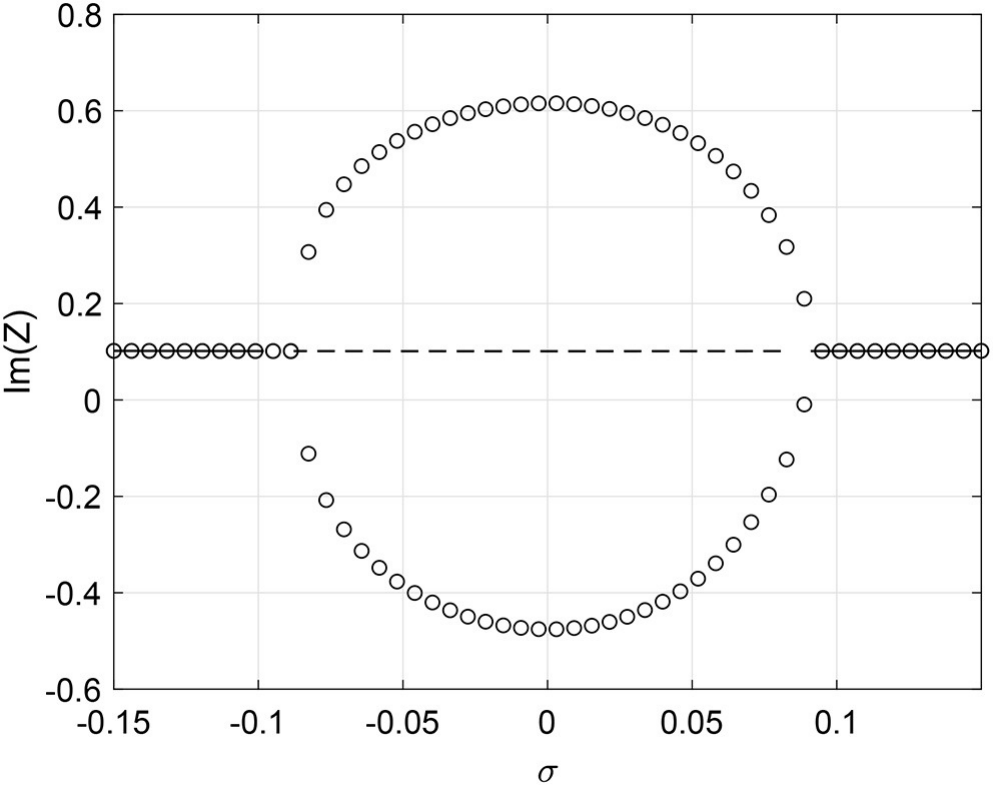
Intrinsic frequency dependence $\omega_{0}\left(k_{out}\right)=1+\sigma {\hat{k}}_{out}$. Solid line: stable fixed point of (44); dashed line: unstable fixed point. Circles: maximum and minimum over one period of Im(*Z*). Other parameters: Δ = 0.05, β = 0, ϵ = 0.2, *m* = 100, *M* = 400.

**Figure 14:**
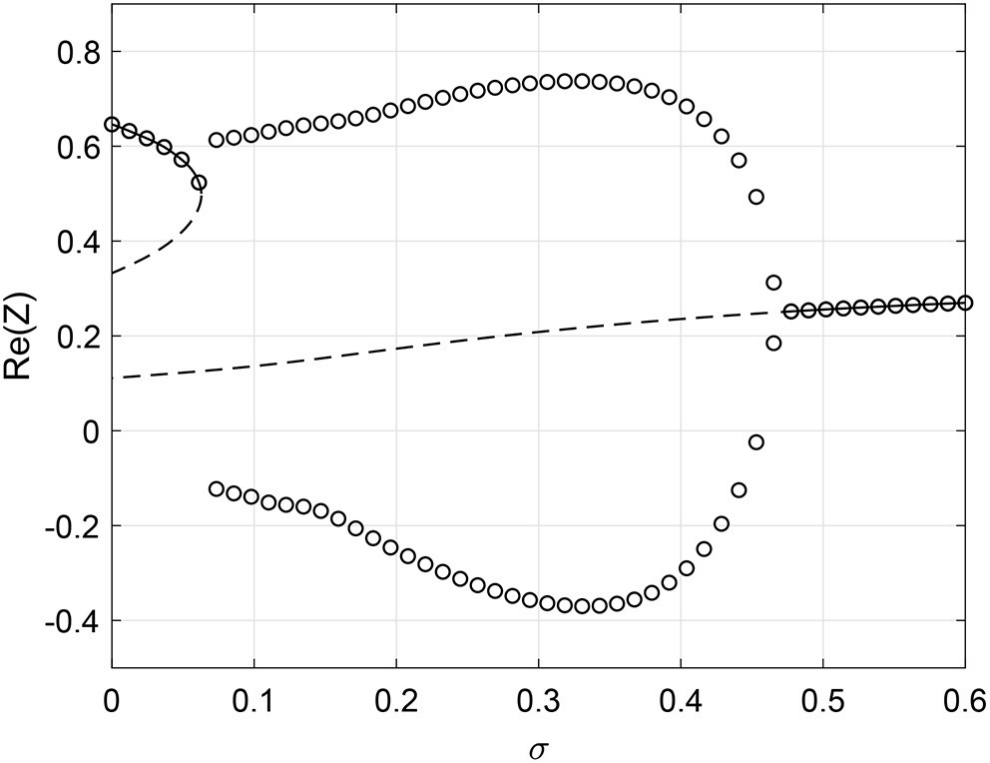
Phase shift dependence $\beta \left(k_{out}\right)=\sigma \left({\hat{k}}_{out}+1\right)$. Solid line: stable fixed point of (44); dashed line: unstable fixed point. Circles: maximum and minimum over one period of Re(*Z*). Other parameters: Δ = 0.05, ω_0_ = 1, ϵ = 0.8, *m* = 100, *M* = 400.

Setting $\omega_{0}\left(k_{out}\right)=1+\sigma {\hat{k}}_{out}$ and varying σ we obtain the results in Figure 13. Two Hopf bifurcations are seen, as in Figure 9A but at different values of σ from in that figure. Writing $\beta \left(k_{out}\right)=\sigma \left({\hat{k}}_{out}+1\right)$ and varying σ we obtain the results in Figure 14. The bifurcations are the same as in Figure 10A, but again, at different values of σ. Using the parameters shown in Figure 11A, setting $\Delta =0.09+\sigma {\hat{k}}_{out}$ and varying σ ∈ [−0.09, 0.09] (as Δ cannot be negative) the fixed point was always stable (not shown). Simulations of a discrete network of *N* = 2, 000 oscillators confirmed all of the results in this section (not shown).

## 6. Parameter Assortativity

We now consider assortativity by a parameter other than degree, in this case ω_0_ value. We first describe how to create a network with such assortativity, then derive the relevant continuum equations. We follow Skardal et al. (2015) in our derivation.

To create a particular network we first create a network where the in- and out-degrees of all oscillators are the same, in order that degree not affect the dynamics. To do this we use the configuration model (Newman, 2003), then remove all self-connections and multi-edges as before. With *N* oscillators we randomly choose *N* *target* values of ω_0_ from a distribution *p*(ω_0_), which is non-zero only if $\omega_{0}\in \left[{\underline{\omega }}_{0},{\bar{\omega }}_{0}\right]$, i.e., ω_0_ is the minimum value of ω_0_ and ${\bar{\omega }}_{0}$ is the maximum, and assign these to oscillators. We can calculate the assortativity of the network using similar ideas as those in section 4. We calculate the Pearson correlation coefficient

(49) $$r=\frac{\sum_{e=1}^{N_{e}}\left(\omega_{0,e}^{\prime }-\langle \omega_{0}^{\prime }\rangle )(\omega_{0,e}-\langle \omega_{0}\rangle \right)}{\sqrt{\sum_{e=1}^{N_{e}}{\left(\omega_{0,e}^{\prime }-\langle \omega_{0}^{\prime }\rangle \right)}^{2}}\;\sqrt{\sum_{e=1}^{N_{e}}{\left(\omega_{0,e}-\langle \omega_{0}\rangle \right)}^{2}}}$$

where $\omega_{0,e}^{\prime }$ is the value of the target ω_0_ associated with the oscillator at the start of edge *e* and ω_0, *e*_ is the value of the target ω_0_ associated with the oscillator at the end of edge *e*, and *N*_*e*_ is the number of edges. The means are

(50) $$\begin{array}{l} \langle \omega_{0}^{\prime }\rangle =\frac{1}{N_{e}}\overset{N_{e}}{\underset{e=1}{\sum }}\omega_{0,e}^{\prime }\;\langle \omega_{0}\rangle =\frac{1}{N_{e}}\overset{N_{e}}{\underset{e=1}{\sum }}\omega_{0,e} \end{array}$$

Initially the network will have *r* ≈ 0. We induce assortativity in a similar way to that described in section 4. We randomly chose two edges, one connecting oscillator *j* to oscillator *i* and another connecting oscillator *l* to oscillator *h*. We calculate their contribution to the numerator of Equation (49) and the contribution if we replaced these two edges with one connecting oscillator *j* to oscillator *h* and another connecting oscillator *l* to oscillator *i*. If performing this swap increases *r* we make the swap, otherwise we do not. We then repeat this process many times, storing *A* and calculating the value of *r* at regular intervals. (To decrease *r* from its initial value of 0 we just consider whether making the swap decreases *r*). As a last step, in order to use the Ott/Antonsen ansatz, we then randomly assign to oscillator *i* a value of ω_*i*_ chosen from a Lorentzian with mean equal to the *target* ω_0_ for that oscillator and with half-width-at-half-maximum Δ. This will result in the creation of a network in which all oscillators have the same in- and out-degree, but those with high ω_0_ are more likely to connect to those also having high ω_0_ and vice versa.

To derive the continuum equations we see that the state of an oscillator can only depend on its ω_0_ value. We discretize the range of ω_0_ values, $\left[{\underline{\omega }}_{0},{\bar{\omega }}_{0}\right]$, into *m* equal-sized bins, and thus we have

(51) $$\begin{array}{l} \frac{db_{s}\left(t\right)}{dt}=\frac{\epsilon e^{-i\beta }R_{s}\left(t\right)}{2}+\left[i\omega_{s}-\Delta +i\epsilon \sin\beta R_{s}\left(t\right)\right]b_{s}\left(t\right)\\ \;-\frac{\epsilon e^{i\beta }R_{s}\left(t\right)}{2}{\left[b_{s}\left(t\right)\right]}^{2} \end{array}$$

for *s* = 1, 2, …*m*, where ω_*s*_ is the value of ω_0_ in the center of the *s*th bin. The analog of Equation (8) is

(52) $$\begin{array}{l} R_{s}\left(t\right)=\frac{1}{\langle k\rangle }\overset{m}{\underset{u=1}{\sum }}E_{su}G_{u}\left(t\right) \end{array}$$

where 〈*k*〉 is the degree of each oscillator,

(53) $$\begin{array}{l} G_{s}\left(t\right)=a_{q}\left[C_{0}+\overset{q}{\underset{j=1}{\sum }}C_{j}\left\{{\left[b_{s}\left(t\right)\right]}^{j}+{\left[{\bar{b}}_{s}\left(t\right)\right]}^{j}\right\}\right] \end{array}$$

and the matrix *E* encodes the connectivity of the network, i.e., *E*_*su*_ is proportional to the number of oscillators in the *u*th bin which connect to oscillators in the *s*th bin, which can be determined from the connectivity matrix *A*. As in section 4, we record *A* at discrete values of the correlation coefficient *r*, so can construct *E*(*r*) at those values. We fit a quadratic through each entry of *E* as a function of *r* and thus write

(54) $$\begin{array}{l} E\left(r\right)=Br^{2}+Cr+D \end{array}$$

where *B, C*, and *D* are *m* × *m* constant matrices.

**Figure 15:**
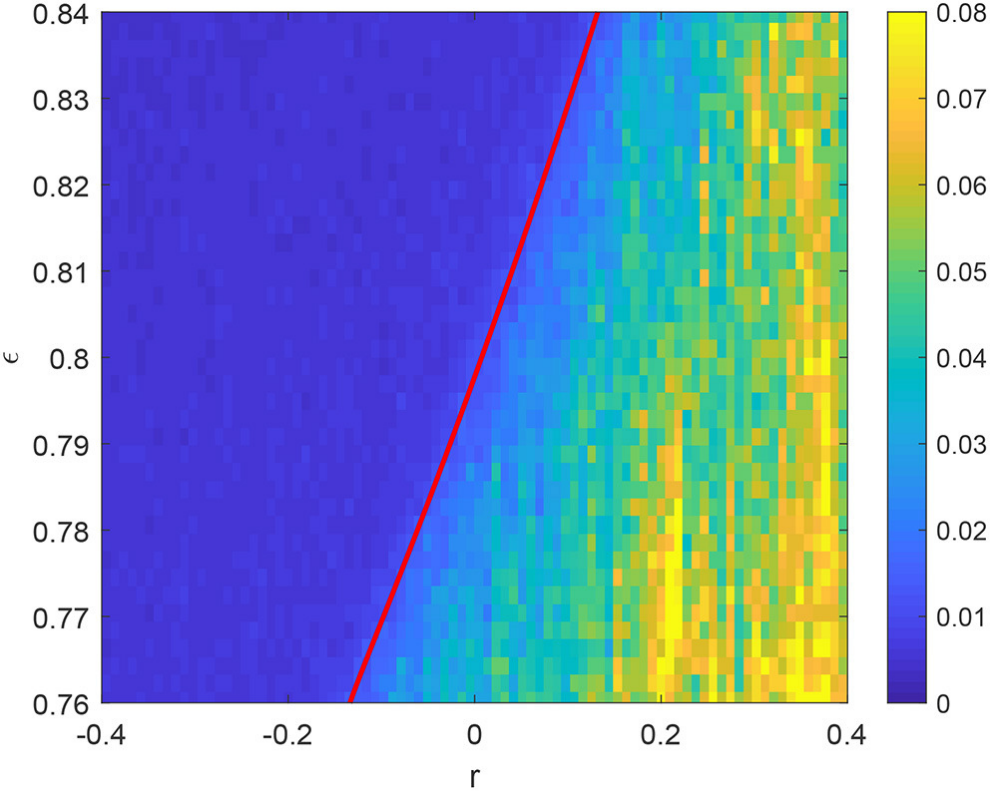
Difference between the maximum of |*Z*| over the last 50 time units out of 100 and the minimum, having already discarded the first 50 as transient. *p*(ω_0_) is uniform on [0, 2]. The red curve shows the Hopf bifurcation of the steady state of (51) which is stable to the left of this curve. Other parameters: β = 0, Δ = 0.01, 〈*k*〉 = 100, *N* = 2, 000. We use *m* = 20 bins to calculate the blue curve.

As an example we choose β = 0, Δ = 0.01, and *p*(ω_0_) to be the uniform distribution on [0, 2]. (*p*(ω_0_) must have bounded support so we can discretize its domain into a finite number of bins.) We compare the results of simulating a full network from Equation (1) with those from the reduced model in Equation (51). We use a network of *N* = 2, 000 with each oscillator having degree 〈*k*〉 = 100. We have stored the connectivity matrix *A* at 101 values of *r*, and vary both ϵ and *r*. At each point in this parameter space we solve Equation (1) for 100 time units, discard the first 50 as transients, then calculate the order parameter using Equation (30). The difference between the maximum of |*Z*| over the final 50 time units and the minimum of |*Z*| over this time is shown in Figure 15.

When this difference is close to zero, most of the oscillators are “locked” at zero frequency, but for ϵ = 0.8 there is a transition at *r* ≈ 0 where some the oscillators start unlocking, with those having largest ω_0_ unlocking first. Note that this is not a “classical” bifurcation, as the system is not at fixed point before this transition. However, solving the reduced Equations (51) we find that there is a stable fixed point to the left of the red curve in Figure 15 which is destroyed in a Hopf bifurcation, leading to periodic and then quasiperiodic behavior as *r* is increased. Thus the reduced model provides an explanation for the observed behavior of the full model (1).

The results in Figure 15 are an example of the types of results we can obtain using the framework presented here. We could vary parameters other than ϵ, or introduce assortativity by another intrinsic parameter, β. In this case we would have to use a different measure of correlation between the β values for connected oscillators, as β is an angular variable (Fisher and Lee, 1983).

## 7. Conclusion

We studied large directed networks of Winfree oscillators under the assumption that the expected dynamics of an oscillator in such a network is determined by its degree: either its in-degree, out-degree, or both (apart from the homogenous degree networks in section 6). Using the Ott/Antonsen ansatz we find that the dynamics are given by Equation (11). Correlations between the in- and out-degree of an oscillator were introduced using a Gaussian copula in section 3, where we investigated the influence of these correlations on the position of bifurcations destroying stable periodic orbits. In section 4, we investigated four types of degree assortativity, as in Bläsche et al. (2020), and found similar results, viz. two types of assortativity have no effect on the network dynamics, while the other two do. Correlations between an oscillator's intrinsic parameter and either its in- or out-degree were examined in section 5. Parameter assortativity was considered in section 6. The framework presented here is quite general, and we believe it to be a powerful method for investigating the general issue of the influence of a network's structure on its dynamics.

The main tool used was numerical continuation and bifurcation analysis of a large number of coupled ODEs (Laing, 2014), which enabled the determination of bifurcation points as parameters were varied—including correlations between various network properties. Following such bifurcations shows the influence of network properties in their dynamics.

The influence of these correlations and assortativities on network synchrony is complex, nuanced, and multi-faceted. Introducing degree correlations within oscillators subtly shapes sensitivity of the network to oscillator parameters such as variability of intrinsic frequencies, Δ, and coupling strength, ϵ. Assortativities between the degrees of connected oscillators can have similar effects for in-degree correlations—r(in,·)—or none at all—r(out,·). More dramatically, for the parameters considered, inducing correlations between oscillator degree (in or out) and intrinsic frequency, ω, destroys oscillatory synchrony. Similarly, if the degree and phase offset, β, are correlated, this may cause or destroy synchronized oscillations. The theme continues with assortativities between intrinsic frequencies, where if they are excessively assortative, oscillators in the network unlock from the population—requiring a higher coupling level to stay locked. Conversely, correlating an oscillator's degree with the width of the distribution from which its intrinsic frequency is chosen, Δ, has little effect.

Network structure such as preferential attachment between similar (or dissimilar) oscillators and the influence we have observed here in idealized systems may reflect structural influences in physiological networks of neurons. Intrinsic connectivity preferences observed of neurons grown in culture—e.g., similar numbers of synaptic or dendritic processes connected to each other in groups—results in strong assortativity patterns (de Santos-Sierra et al., 2014; Teller et al., 2014) further inferred in the human cerebral cortex (Hagmann et al., 2008). Our observations of network structure influencing the overall synchrony of a network may be a structural means of calibrating the dynamics of physiological neurons.

Regarding section 5, correlating degree with intrinsic frequency is known to cause explosive synchronization, characterized by bistability between asynchronous and partially synchronized states, in undirected networks of Kuramoto phase oscillators (Gómez-Gardeñes et al., 2011; Liu et al., 2013). We did not observe such behavior but we only considered uniform degree distributions (not power law; Gómez-Gardeñes et al., 2011; Liu et al., 2013) and have directed connections, not undirected. Also, there are many ways to correlate an intrinsic parameter with a degree (Skardal et al., 2013); our form of modification keeps the parameter for nodes with mean degree the same and increases/decreases the parameter for those with degrees above/below mean (or vice versa) in a linear way.

We certainly do not yet have a full understanding of the possible dynamics of the network (1). Possible extensions of the work here include simultaneously having more than one type of structure present in the network (for example, both within-oscillator degree correlations *and* degree assortativity) or correlating an oscillator's intrinsic parameter with some other network property such as the oscillator's centrality (Newman, 2018) or local clustering coefficient (Watts and Strogatz, 1998). More detailed knowledge about the connectivity in networks of neurons of interest would provide motivation to study these extensions, and help verify some of our results.

## Data Availability Statement

The raw data supporting the conclusions of this article will be made available by the authors, without undue reservation.

## Author Contributions

All authors listed have made a substantial, direct and intellectual contribution to the work, and approved it for publication.

## Conflict of Interest

The authors declare that the research was conducted in the absence of any commercial or financial relationships that could be construed as a potential conflict of interest.
